# To Be or Not to Be a Pathogen: *Candida albicans* and Celiac Disease

**DOI:** 10.3389/fimmu.2019.02844

**Published:** 2019-12-05

**Authors:** Giorgia Renga, Marina M. Bellet, Claudia Stincardini, Marilena Pariano, Vasilis Oikonomou, Valeria R. Villella, Stefano Brancorsini, Carlo Clerici, Luigina Romani, Claudio Costantini

**Affiliations:** ^1^Department of Experimental Medicine, University of Perugia, Perugia, Italy; ^2^Division of Genetics and Cell Biology, European Institute for Research in Cystic Fibrosis, San Raffaele Scientific Institute, Milan, Italy; ^3^Gastroenterology Unit, Santa Maria della Misericordia Hospital of Perugia, Perugia, Italy

**Keywords:** celiac disease, *Candida albicans*, mast cells, IL-9, tryptophan, immune tolerance

## Abstract

Celiac disease (CD) is an immune-mediated disorder triggered by the ingestion of gluten and characterized by reversible small-bowel mucosal atrophy in genetically predisposed subjects. Although the prevalence of CD has increased, many aspects of this pathology are still unrecognized. *Candida albicans*, a commensal of the human gastrointestinal tract, has been linked to CD for a long time based, among others, upon the observation of similarity between the fungal wall component, hyphal wall protein 1, and CD-related gliadin T-cell epitopes. We have recently demonstrated that *Candida* may switch from commensal to pathogen contingent upon several players, including mast cells, key sentinels of the immune system at the interface between the environment and the host, and the pleiotropic cytokine IL-9. However, other factors are likely to play a role by altering the balance between inflammation and tolerance. In this regard, tryptophan and its metabolites are increasingly being recognized in promoting mucosal homeostasis by balancing the immune response to external cues. Based on these premises, we will discuss how the output of *Candida* colonization in the gut is highly contextual, being determined at the intersection of many immunological (IL-9/mast cells) and metabolic (tryptophan) pathways that ultimately dictate the *Candida* commensalism vs. pathogenicity in CD, thus paving the way for novel therapeutic opportunities in CD.

## Introduction

Celiac disease (CD) is an immune-mediated disorder triggered by the ingestion of gluten, a major protein in wheat, and characterized by reversible small-bowel mucosal atrophy in genetically predisposed subjects. CD affects about 1% of people in most populations but evidence suggests that the prevalence is increasing. Although the prevalence of celiac disease has increased, this pathology is still unrecognized. CD symptoms can appear from childhood to senior adulthood although the average age of diagnosis is between the 4th and 6th decades of life, indicating an important role of age in the onset of the disease. An early diagnosis is important to reduce the intestinal and extra-intestinal damages due to the recurrent gluten exposure. Unfortunately, it has been reported that for each clinical diagnosis of CD, an average of 5 to 10 individuals remains undiagnosed. The failure to diagnosis CD is usually due to atypical, minimal or even absent symptoms (1). The patients presenting malabsorption, weight loss, and steatorrhea are a small proportion of the total population affected by CD. For this reason, over 10 years ago, Ferguson et al. compared CD to an iceberg in which symptomatic patients represent the visible peak and asymptomatic subjects the invisible part of the floating rock (2). Interestingly, several studies have indicated that CD is diagnosed predominantly in women and this is partially due to increased prevalence in women relative to men, to the fact that women use healthcare services more than men and that women have the highest risk to develop autoimmune conditions (3–5). As a matter of fact, the symptoms of CD in women are more severe and rapid than in men (6).

The major environmental factor for the development of CD is gliadin, a monomeric protein contained in the gluten, rich in glutamine and proline. This chemical structure renders part of the molecule resistant to proteolytic degradation by gastrointestinal enzymes, leaving large peptides up to 33 amino acids long (7). In healthy people, most of these peptides are simply excreted without triggering the immune response. On the contrary, in CD subjects the deamidation of peptides by the enzyme tissue transglutaminase 2 (tTG2) increases the immunogenicity of gliadin, favoring the binding to the HLA-DQ2 or HLA-DQ8 molecules situated on antigen presenting cells. This leads to the exaggerated activation of the adaptive immune response mediated by CD4^+^ T cells (8, 9). Interestingly, only a small portion of individuals with at-risk HLA exposed to gluten develop CD, suggesting that both factors are necessary but not sufficient for developing CD (10). Thus, others environmental factors are possibly involved in the switches of the tolerance–immune activation balance in CD. Among these, the spectrum of intestinal microorganisms and how they change over time are considered to be important (11). In this regard, the similarity between the hyphal wall protein 1 (Hwp1) of *Candida albicans*, the major fungal commensal residing in the human gastrointestinal tract and two CD-related gliadin T-cell epitopes (12, 13), the humoral cross-reactivity between Hwp1 and gliadin (14) and the high fecal counts of *Candida* yeasts observed in CD patients (15) have all been taken to implicate *Candida* in the pathogenesis of CD.

Herein, we will discuss how the output of *Candida* colonization in the gut is highly contextual, being determined at the intersection of many immunological (IL-9/mast cells) and metabolic (tryptophan) pathways that ultimately dictate the *Candida* commensalism vs. pathogenicity in CD.

## Mast Cells: When Sentinels Become Inflammatory Culprits in CD

Although CD is considered a T cell-mediated enteropathy, there is growing evidence supporting the crucial role of innate immunity in the development of CD (16). It is therefore critical to study the role of innate immunity in the inductive and effector phases of disease in order to understand the disease as a whole. Mast cells (MCs) are tissue-resident cells typically located at the strategical location involved in host defense and belong to the innate immune system. MCs are abundant in the gastrointestinal tract and consist ~2–3% within the lamina propria in healthy individuals (17). The ever-changing environment characteristics of the intestinal tract contribute to MC switching phenotypes, a transdifferentiation process by which MCs synthesize and release specific mediators depending on the environment, so influencing their unique ability to regulate homeostasis or promote inflammatory processes (18). Thus, MCs are viewed as important sentinels in host defense against bacterial, viral and parasitic infections but also as promoters of several gastrointestinal diseases such as food allergy (19, 20). Based on the protease expression, MCs can be distinguished in mice into mucosal-type MCs (MMCs), located in mucosal compartments and expressing the proteases chymase, and to connective tissue-type MCs (CTMCs), located in submucosa and expressing the proteases chymase, tryptase and carboxypeptidase A (21). Several studies have highlighted that aberrant MC activation is associated with increased intestinal permeability and inflammation (22). MC-derived tryptase induces acute intestinal inflammatory responses by activating a protease-activated receptor 2 expressed on the intestinal epithelial cells (23). This activation leads to a redistribution of zonulin and occludin, two important proteins that ensure the integrity of intestinal barrier, resulting in increased permeability (24).

Because of the ability to disrupt intestinal epithelial barrier, MCs are known to promote inflammation upon repeated exposure of ingested antigen. In 2015, Chen et al. demonstrated that MMCs considerably expand after repeated exposure to ingested antigens, thus favoring food allergic response and systemic anaphylaxis (25). Of interest, these cells can secrete prodigious amount of IL-9, a pleiotropic cytokine produced by both innate and adaptive immunity cells (26), suggesting that IL-9 and MCs may synergize to develop food allergy. In fact, mice ablated of MMC-IL-9-dependent cells failed to develop intestinal mastocytosis, which resulted in decreased food allergy symptoms promptly restored by the adoptive transfer of these cells (25). The dual capability of MCs to both promote epithelial damage and aggravate food allergy symptoms may result destructive in CD. Several reports documented an increased MC number in the untreated CD subjects that returns to normal levels after gluten withdrawal. Conversely, others showed a lower MC number in intestinal biopsies from untreated CD patients compared to healthy subjects, which return to the normal range in patients subjected to a gluten-free diet (27). More recently, 20 subjects with non-celiac gluten sensitivity and 16 CD patients were enrolled to evaluate the expression of specific markers of the innate immune system and have shown an increased MC accumulation in the intestinal mucosa on both groups (28). In addition, Frossi et al. have found that the increased density of infiltrating MCs in CD intestinal biopsies correlates with an increased inflammatory grade, according to the Marsh classification, and that, by activating the MyD88 pathways, MCs release *in vitro* inflammatory cytokines and skew myeloid populations toward a Th1-polarizing environment (29).

All in all, these results indicate that MC plasticity may be a double-edged sword in CD and as-yet uncharacterized environmental changes can drive MCs to exacerbate mucosal inflammation by impairing barrier integrity and promoting food allergy.

## *Candida albicans*: to be or not to be a Pathogen in CD

*C. albicans* is a human commensal with an extraordinary ability to well-adapt for growth in the gastrointestinal tract because of a delicate interplay between host immunity, the microbiota, and the fungus (30–32). The ability to survive as a commensal in several anatomically distinct sites, each with its own specific set of environmental pressures, and switch to pathobiont in particular conditions is at the base of *Candida* virulence. The transition between yeast and hyphal morphologies is thought to underlie much of the variation in virulence observed in several host tissues (30).

As already mentioned, the hypothesis that *C. albicans* may be a trigger in CD has been proposed after the observation of similarity between Hwp1 and two CD-related gliadin T-cell epitopes. The high homology allows tissue TG2 to covalently link fungal protein and enable *Candida* to adhere strongly to the intestinal epithelium (12, 33). This hypothesis has been supported by the finding of higher levels of anti-Hwp1, anti-gliadin and anti-TG2 antibodies in the serum of CD patients more than healthy controls, suggesting that the presence of *Candida* in CD may activate an unstrained immune response that, at least in susceptible individuals, may lead to increased inflammation and the characteristic villous atrophy (14). In addition, Harnett et al. were able to detect *Candida* sp. in 33% of CD fecal specimens compared to 0% of the control group confirming the idea that *Candida* may act as a trigger of autoimmune responses in genetically predisposed subjects (15). Thus, fungal components might potentially contribute to CD pathogenesis by modifying immunogenic epitopes of gluten and ensuing immune response. However, *C. albicans* is well-adapted for growth in the gastrointestinal tract where inflammation may perturb the resident bacterial community creating conditions that favor *Candida* colonization and inflammation. In fact, despite being implicated in intestinal immunopathology and sensitization to food antigens (34), *C. albicans* colonization also protects against local (31) and distant (35) immune pathologies in mice (36, 37).

Collectively, our current knowledge on the behavior of *C. albicans* in the gastrointestinal tract depicts a scenario in which the environmental conditions coupled to the individual genetic susceptibility impacts on the relationship between the host and *C. albicans* that may be either protective or pathogenic. Sorting out the key factors that tip the balance in either direction may be instrumental for future therapeutic approaches.

## IL-9 and *Candida*: a Fatal Duo in CD

Growth factors, cytokines, intestinal bacteria, dietary components, and proteases are known to regulate the intestinal barrier function. Particularly, cytokines can alter directly or indirectly the intestinal permeability by both influencing the structure and function of the tight junctions, multi-protein complexes that seal the space between adjacent cells (38, 39), and favoring the commensal-to-pathogen transition of gut microbes (40–42). This seems to be the case for IL-9 known to be able to regulate the intestinal barrier function. Indeed, it has been demonstrated that IL-9-deficient mice fail to develop experimental oral antigen-induced intestinal anaphylaxis. On the contrary, the IL-9 overexpression predisposed to oral antigen sensitization and increased the intestinal permeability (43). In addition, Gerlach et al. showed that IL-9 deficiency reduces inflammatory parameters in acute murine colitis by influencing the expression of occludin and claudin-1 (44). The ability to impair barrier function and predispose to food allergy points to IL-9 as a critical determinant in the predisposition to several gastrointestinal diseases such as CD.

Beyond its ability to affect the intestinal epithelial barrier, IL-9 acts on *Candida* behavior. We recently demonstrated that IL-9 and MMCs induce an inflammatory status that promotes barrier function loss and fungal dissemination. This condition favoring *Candida* transition from commensal to pathobiont may be dangerous when a proper adaptive immune response does not occur as in the case of CD (45). As expected, we observed an increased IL-9 positivity in the duodenal biopsies from CD patients that correlated with enhanced inflammation as well as an increased susceptibility of gluten-sensitized mice upon infection with the fungus (45). These results suggest that the IL-9/MC axis could be an attractive drugable pathway to prevent the clinical unwanted consequences of fungal colonization. However, the occurrence of *Candida* pneumonia in patients receiving imatinib mesylate treatment suggests that MC-targeted therapy might predispose patients to opportunistic and life-threatening fungal infections (46). We have provided a plausible explanation for MC-mediated protection in the gut. Consistent with their immunoregulatory role on adaptive immune responses, tissue remodeling, homeostasis and peripheral tolerance (47, 48), CTMC promoted intestinal immune tolerance *via* the activation of different tryptophan (trp) metabolic pathways (45). We have found that IL-9 activates CTMC for local immune tolerance via both the indoleamine 2,3-dioxygenase (IDO)1 and the trp hydroxylase 1 (TPH1) enzymatic pathways (45). Thus, the relative high levels of IL-9 and MMC observed in biopsies from patients with CD may promote an inflammation-driven intestinal dysbiosis to which trp deficiency may contribute. As microbiota perturbation was observed in condition of IL-9 or MC deficiency (45), we concluded that the IL-9/MC axis, by integrating signals derived from the perturbed host/microbiota homeostasis, might act as signature that discriminates between the pathogenic vs. protective role of the fungus in the gut.

In conclusion, IL-9 may play a dual action in the gastrointestinal tract and, similarly to MCs and *Candida*, to which IL-9 is linked by an intricate cross-talk, opposite outcomes may come out, that could either result in protection, with the induction of a tolerogenic state, or disease, with inflammation and *Candida* transition to a pathogenic state.

## The Trp Metabolic Pathways: New Players in CD

Oral tolerance is a specific type of peripheral tolerance that prevents hypersensitivity reactions induced by the exposure of antigen (49) and is defective in CD. Different mechanisms mediate development and expression of tolerance and among these, the trp metabolic pathways are highly relevant. Trp is an essential aromatic amino acid that human beings obtain through diet. Dietary trp can directly be converted by host IDO1 or TPH1 enzymes into kynurenines (50) or serotonin (5-HT) (51), respectively, and by gut microbiota into ligands for aryl hydrocarbon receptor (AhR) (52). Thus, the host and the microbiota influence the trp catabolism and are critical inducers of regulatory responses in the gut (53, 54). A breakdown in regulatory mechanisms results in a set of chronic inflammatory conditions such as in CD (55). Several studies have reported a microbial dysbiosis in CD. Indeed, CD patients show a lower rate of beneficial to harmful bacteria compared to healthy controls and this is probably due to inherited differences in the expression of carbohydrates (56). The altered intestinal microbiota composition observed in CD led to increased IDO1 expression in myeloid cells of the intestinal lamina propria with increased levels of kynurenines (57, 58). Kynurenines play a tolerogenic role by promoting regulatory T cells (Tregs) (59). In CD, there are conflicting studies on Tregs function. Some studies indicated that Tregs have impaired suppressive function and this might cause loss of tolerance to gluten, but also to self-antigens (60, 61), while others reported a higher density of CD4^+^CD25^+^Foxp3^+^ T cells in duodenal biopsies of active CD patients compared to treated CD and control group. Despite the massive recruitment of Tregs *in situ* by gliadin, the authors described an ineffective control on CD4^+^ effector T cells by Tregs, likely due to the blocking function of IL-15, IL-21, and IFN-γ in CD subjects (62–64).

The inability of Tregs to control inflammatory response may be also due to the defective AhR signaling observed in CD. AhR is a ligand-dependent transcription factor that senses both xenobiotic and endogenous ligands, including microbiota-derived factors, and regulates immune homeostasis in the gut through IL-22 (65). The expansion of Tregs is a process that, at least partially, also requires the activation of AhR. In fact, kynurenines act as ligand for AhR and this leads AhR to translocate into the nucleus where it induces the expression of Foxp3 (59). Recently, Dinallo et al. documented a diminished AhR expression in the intestinal mucosa of patients with active CD as compared with inactive CD patients and normal controls suggesting that defective AhR signaling could contribute to amplify detrimental immune signals in CD mucosa (66). As most AhR ligands in the gut are of microbial origin (67), this directly links gut microbial dysbiosis to loss of immune tolerance in CD.

Beside the kynurenine pathway, trp degradation leads to the production of 5-HT. 5-HT is a neurotransmitter involved in the regulation of numerous biological functions. While 5-HT is known to act primarily on the brain, close to 95% is synthesized and released by the enterochromaffin cells of the gut, in which 5-HT maintains host defense. Many evidences link depression and gluten intolerance to serotonergic deficiency (68, 69). A study on rats showed that brain 5-HT levels decreased after the rats were fed wheat (70). Another study found that the majority of adolescents with CD display depressive symptoms before the diagnosis of CD and have low free trp levels. After conducting a gluten-free diet, CD subjects showed the improvement of depressive symptoms and increased levels of free trp levels (71). After 1 year on a gluten-free diet, patients experienced a significant increase in 5-HT (72). By contrast, other studies observed an increased level of 5-HT in CD. In 2006, Coleman et al. observed an increased number of ECs and significantly high peak level of 5-HT both in plasma and in duodenum of CD subjects (73). In addition, Di Sabatino et al. reported a significantly increase of ECs and 5-HT in the gut of refractory CD (74).

Trp metabolism plays a pivotal role also in the regulation of the immune response against *C. albicans*. On the one hand, the microbial-dependent AhR/IL-22 axis controls the initial fungal growth and infectivity at mucosal surfaces (52); on the other, host IDO1 and kynurenines prevent dysregulated immunity caused by *Candida* (75). In addition, the interaction between 5-HT and *Candida* may diminish the virulence properties of the fungus suggesting an important role also for 5-HT in antifungal host defense (76).

The activation of trp metabolic pathways and the generation of bioactive molecules with a role in maintaining mucosal homeostasis in the gastrointestinal tract—in terms of microbial eubiosis, immune tolerance, and control of *Candida* virulence—are emerging as key players in gut health. Their dysregulation may play a causative role in CD thus representing potential targets for therapeutic strategies.

## Conclusion

**Figure 1:**
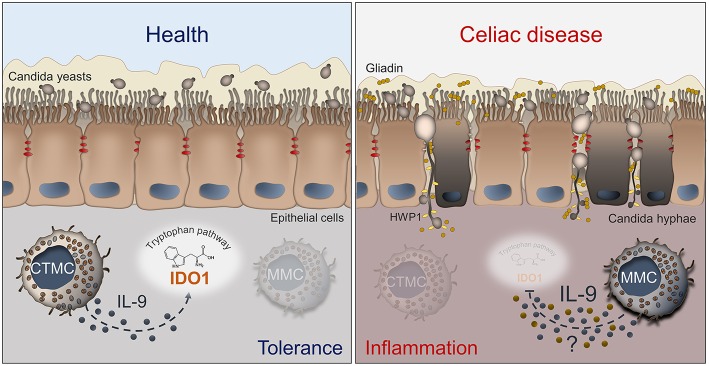
Schematic depiction of the interactions between *Candida*, MCs and IL-9 in celiac disease. The left panel shows that, in healthy conditions, *C. albicans* is commensal in the gut and mucosal homeostasis is maintained by CTMCs that, by producing low levels of IL-9 (blue dots), sustain IDO1 activity and tolerance. In the right panel, what may happen in celiac disease is depicted. The barrier integrity is disrupted and *Candida* switches from the yeast to the hyphal form and crosses the epithelial lining, thus contributing to tissue damage. The pathogenic switch of *Candida* is favored by the predominance of MMCs that inhibit the activity of IDO1 by producing high levels of IL-9 and likely other inflammatory factors (yellow dots). Details are described in the text. CTMC, connective tissue-type mast cells; IDO1, indoleamine 2,3-dioxygenase; HWP1, hyphal wall protein; MMC, mucosal-type mast cells.

Despite increasing knowledge about CD, further avenues of investigation are necessary to better understand the pathogenesis and improve the treatment of patients with this condition. For a long time, *C. albicans* has been proposed as a possible trigger in CD. However, our recent study suggests a more intriguing role of *C. albicans* at the host/microbiota interface in CD (Figure 1). A better definition of this model might provide a unique opportunity for: (i) rationale antifungal therapy in CD by stratifying patients according to their immune and metabolic risk to develop a pathological *Candida* infection; (ii) use of IL-9 inhibitors to restore epithelial homeostasis in condition of inflammatory and dysbiotic damage; (iii) develop novel therapeutics by resorting to postbiotics that at variance from antibiotics maintain gut health in accordance with the local microbiota.

## Author Contributions

VO designed the figure. GR, LR, and CC wrote the paper. All authors have made a substantial, direct and intellectual contribution to the work, and approved it for publication.

### Conflict of Interest

The authors declare that the research was conducted in the absence of any commercial or financial relationships that could be construed as a potential conflict of interest.
